# Alterations on magnetic resonance imaging of the neonatal brain: correlations with prenatal risk factors and transfontanellar ultrasound findings

**DOI:** 10.1590/0100-3984.2021.0149-en

**Published:** 2022

**Authors:** Jéssica Tedesco Sartori, Luciana Estacia Ambros, Giordana Isabela Siqueira Callegaro

**Affiliations:** 1 Universidade Federal da Fronteira Sul (UFFS), Passo Fundo, RS, Brazil.; 2 Hospital São Vicente de Paulo (HSVP), Passo Fundo, RS, Brazil.; 3 Universidade de Passo Fundo (UPF), Passo Fundo, RS, Brazil.

**Keywords:** Neuroimaging, Ultrasonography, Magnetic resonance imaging, Infant, newborn, diseases, Neuroimagem, Ultrassonografia, Ressonância magnética, Doenças do recém-nascido

## Abstract

**Objective:**

To describe the alterations seen on magnetic resonance imaging (MRI) of the brain in newborns, correlating those alterations with the transfontanellar ultrasound (TFUS) findings, and to describe the main risk factors identified.

**Materials and Methods:**

We evaluated the examinations of 51 patients who were submitted to brain MRI with a neonatal protocol during hospitalization. We evaluated the MRI findings and correlated them with previous TFUS findings, using the last TFUS performed in order to minimize the risk of bias. Data were obtained from medical records, and the images were reviewed by a radiologist specializing in neuroimaging.

**Results:**

Of the 51 patients evaluated, 21 (41.2%) were extremely preterm infants and 22 (43.1%) were extremely-low-birth-weight infants. Alterations were seen on 16 (31.4%) of the TFUS examinations and on 30 (58.8%) of the brain MRI scans, the most common finding being germinal matrix hemorrhage. The positive and negative predictive values of TFUS in relation to MRI were 87% and 54%, respectively.

**Conclusion:**

Because TFUS proved to be capable of distinguishing mild and moderate (grade I and II) germinal matrix hemorrhage from the severe forms (grades III and IV), it can be considered a good tool for screening and follow-up, especially in infants with severe disease and risk factors.

## Abstract (pt)

**Objetivo:**

Avaliar alterações encontradas nas ressonâncias magnéticas (RMs) encefálicas neonatais, correlacionando com a ultrassonografia transfontanelar (USTF), e descrever os principais fatores de risco encontrados.

**Materiais e Métodos:**

Foram avaliados exames de 51 pacientes que realizaram RM utilizando protocolo neonatal durante internação hospitalar, correlacionando com resultados da USTF prévia, sendo utilizada, para minimizar as chances de viés, a última USTF realizada. Os dados foram obtidos de prontuário médico e as imagens foram revisadas por médico radiologista especialista em neuroimagem.

**Resultados:**

A população foi composta majoritariamente de recém-nascidos prematuros extremos (21; 41,2%) e de extremo baixo peso (22; 43,1%). Foram encontradas alterações em 16 (31,4%) das USTFs e em 30 (58,8%) das RMs, sendo a hemorragia da matriz germinativa o achado mais frequente. Os valores preditivos positivo e negativo da USTF em relação à RM foram de 87% e 54%, respectivamente.

**Conclusão:**

A USTF mostrou-se importante na distinção entre os graus de hemorragia da matriz germinativa leve e moderada (I e II) dos graus acentuados (III e IV), sendo considerada um bom exame de rastreio e acompanhamento, principalmente em pacientes mais graves e com fatores de risco.

## INTRODUCTION

Advances in medicine have allowed newborns to be considered viable at increasingly lower gestational ages. That has led to various complications, including neonatal intracranial hemorrhage, which is considered the most common acquired structural lesion in this context^(1)^ and is a leading cause of neurological morbidity, especially in preterm infants^(2)^.

Worldwide, approximately 15 million infants are born prematurely each year, corresponding to 11.1% of all live births. Brazil ranks tenth in the number of preterm births, accounting for 279,300 such births in 2010. Complications related to preterm birth are the leading cause of mortality in children under five years of age, and most of these deaths could be avoided with improved neonatal support, mainly in low-income countries^(3,4)^.

The germinal matrix shows a peak of greater development between the 8th and 28th weeks of gestation, with a tendency to involute thereafter, being the site of origin of approximately 90% of intracranial hemorrhages in the neonatal period. Germinal matrix hemorrhage (GMH) occurs most commonly in the caudothalamic groove, which is the last area of the germinal matrix to involute^(4)^.

Among the consequences of neonatal intracranial hemorrhages, the most worrisome are periventricular leukomalacia, encephalomalacia, white matter hemorrhage, posthemorrhagic ventriculomegaly, ventricular dilatation, porencephaly, and altered brain volume, all of which are major causes of persistent neurological deficits, correlating with learning difficulties, cerebral palsy, epilepsy, and other disorders^(5,6)^.

In neonates, the risk for the development of intracranial lesions is associated with factors inherent to pregnancy, childbirth, and the fetus itself. Among the main factors related to the fetus are low gestational age, low birth weight, low Apgar scores, male gender, respiratory distress, the need for resuscitation or endotracheal intubation, metabolic acidosis, sepsis, and premature rupture of membranes^(5)^. The pathogenesis is complex and is probably related to changes in cerebral ischemia/reperfusion, impaired regulation of cerebral blood flow, and inflammatory mechanisms associated with maternal or fetal infection^(7)^.

The classification system most widely used for grading GMHs (and other types of intracranial hemorrhage) is that devised by Papile et al.^(8)^, in which the classifications range from grade I (least severe) to grade IV (most severe). In that system, the GMH grades are defined as follows: grade I, minimal or no extension into the ventricles; grade II, extension into the ventricles but no ventricular dilatation; grade III, extension into the ventricles with ventricular dilatation; grade IV, intraventricular hemorrhage with parenchymal hemorrhage. Most GMHs classified as grade I or II resolve spontaneously, whereas patients with GMHs classified as grade III or IV are more likely to evolve to progressive hydrocephalus, permanent sequelae, and death. In addition, the recognition and adequate assessment of other neonatal hemorrhages, such as extraaxial and intraparenchymal hemorrhages, is essential^(1)^.

Transfontanellar ultrasound (TFUS) is widely used in order to identify abnormalities in preterm newborns at risk of brain injury and impaired neurological development^(9)^, because most such abnormalities are found in asymptomatic newborns. Therefore, screening with TFUS is important, especially in the most vulnerable subgroups^(10)^.

The use of magnetic resonance imaging (MRI) in newborns is still under discussion, the modality mainly being used for the accurate detection of white matter lesions in cases in which the TFUS findings are inconclusive^(6)^. In comparison with TFUS, MRI is more sensitive for the detection of white matter abnormalities, which have been associated with disturbances in brain maturation, as well as neuromotor and developmental impairment. In addition, MRI can assess cerebellar lesions, which may also be associated with a higher risk of neurological abnormalities^(11)^. Despite those advantages, there is as yet no consensus regarding which neuroimaging examination should be performed, when it should be performed, and what its prognostic value is^(11-15)^.

The present study aimed to evaluate the positive and negative predictive values (PPV and NPV, respectively) of neonatal TFUS, in comparison with neonatal brain MRI, for the detection of intracranial hemorrhage, hydrocephalus, and leukomalacia in newborns at a tertiary referral hospital for high-risk pregnancy, as well as to describe the main risk factors found in the study population.

## MATERIALS AND METHODS

This was a cross-sectional, retrospective study of clinical data collected from the medical records of patients 0-6 months of age who were born at or transferred (within the first 30 days of life) to a tertiary referral hospital for high-risk pregnancy between January 2016 and March 2019. The patients selected had undergone brain MRI with a neonatal protocol during hospitalization. Patients with suboptimal brain MRI scans that did not allow adequate assessment were excluded, as were those for whom the medical records were incomplete. The study was approved by the research ethics committee of the institution and registered at Plataforma Brasil (CAAE no. 28628820.4.0000.5342).

Patient data were obtained exclusively from medical records. The neonatal MRI protocol was composed of the following sequences: sagittal T1-weighted; axial T1-weighted; axial T2-weighted; axial fluid-attenuated inversion recovery; axial susceptibility-weighted; axial diffusion-weighted; and axial apparent diffusion coefficient mapping.

The selected patients were evaluated regarding risk factors for neonatal intracranial hemorrhage. Those factors included previous maternal risk factors; prenatal care; complications during the prenatal, perinatal, and postnatal periods; and neonatal risk factors.

The TFUS data were extracted from the radiology reports. The MRI data were also obtained from the radiology reports, and the images were reviewed by a radiologist specializing in neuroimaging. The mean time between TFUS and MRI was 19.5 days (range, 0-63 days).

The TFUS and MRI findings were classified regarding the presence or absence of hemorrhage, leukoencephalomalacia, hydrocephalus, and other pathological changes, as well as the perceived Papile grade (I, II, III, or IV). Because TFUS is a routine test in preterm newborns, the result of the last TFUS performed prior to MRI was considered for evaluation purposes.

A database was built with Microsoft Excel. Statistical analyses were performed with the IBM SPSS Statistics software package, version 22.0 for Windows (IBM Corporation, Armonk, NY, USA).

## RESULTS

Of a total of 52 newborns who were included in the study, one was excluded for having been transferred to the institution more than 30 days after birth. Therefore, the sample comprised 51 newborns. All of the patients had been submitted to the neonatal MRI protocol under anesthesia. Of the 51 examinations evaluated, 35 were performed in a 1.5-T scanner and 16 were performed in a 3.0-T scanner.

Among the mother-infant pairs studied, the mean gestational age at birth was 205.4 days (approximately 29 weeks and 3 days) and there were nine sets of twins (17,6%). Of the 51 newborns evaluated, 25 (49%) were female and 26 (51%) were male. Table 1 describes the maternal and fetal characteristics.

**Table 1:** Maternal and fetal characteristics.

Characteristic	Mean	SD	Min.	Max.
Maternal				
Age (years)	27.98	7.27	16	46
Prenatal care consultations (n)	4.69	2.48	0	13
Fetal				
Gestational age (days)	205.45	23.92	174	273
Birth weight (g)	1.274.51	632.75	485	3,250
1-min Apgar score	5.06	—	0	9
5-min Apgar score	7.24	—	2	10
Length of hospital stay (days)	88.69	71.27	21	531
Length of stay in neonatal intensive care unit (days)	66.71	31.93	12	143

SD, standard deviation; Min., minimum; Max., maximum.

The patients were stratified by gestational age and birth weight^(16)^, as described in Table 2. The sample was composed mainly of extremely preterm, extremely-low-birth-weight infants; there were no overweight and postterm newborns.

**Table 2:** Categorization of newborns.

Variable	(N = 51)
Gestational age, n (%)	
< 28 weeks (extremely preterm)	21 (41.2)
28 — < 32 weeks (very preterm)	18 (35.3)
32 — < 37 weeks (moderate or late preterm)	9 (17.6)
37 — < 42 weeks (term)	3 (5.9)
Birth weight, n (%)	
< 1,000 g (extremely low)	22 (43.1)
1,000 — 1,499 g (very low)	16 (31.4)
1,500 — 2,499 g (low)	9 (17.6)
2,500 — 3,999 g (normal)	4 (7.8)

As can be seen in Table 3, the most common complication of pregnancy was preterm labor, which occurred in 21 cases (41.2%). Of the 51 newborns, 42 (82.4%) required resuscitation in the delivery room. A morphological change was detected during prenatal care in only one case (2.0%). In that case, there was a maternal history of toxoplasmosis in the second trimester of pregnancy and the infant was diagnosed with hydrocephalus.

**Table 3:** Gestational and neonatal complications and risk factors.

Complication/risk factor	(N = 51)
Gestational, n (%)	
Premature labor	21 (41.2)
Premature rupture of membranes	11 (21.6)
Intrauterine growth restriction	9 (17.6)
Chorioamnionitis	8 (15.7)
Oligohydramnios	5 (9.8)
Hypertension	4 (7.8)
Fetal distress	4 (7.8)
Preeclampsia/eclampsia/HELLP syndrome	3 (5.9)
Smoking	3 (5.9)
Neonatal, n (%)	
Resuscitation	42 (82.4)
Endotracheal intubação	39 (76.5)
Sepsis (early + late)	39 (76.5)
Sepsis (early)	26 (51,0)
Cardiopulmonary arrest	4 (7.8)
Anoxia	3 (5.9)

HELLP, hemolysis, elevated liver enzymes, and low platelets.

Table 4 details the imaging findings. The most common finding was intracranial hemorrhage, which was detected in eight TFUS examinations and on 22 MRI scans. In three cases, intraparenchymal hemorrhages detected on MRI were not detected on TFUS.

**Table 4:** Radiological findings.

Modality/finding	(N = 51)
MRI, n (%)				
Any alteration*	30 (58.8)
Hemorrhage	22 (43.1)
Grade I GMH	7 (13.7)
Grade II GMH	10 (19.6)
Grade III GMH	—
Grade IV GMH	2 (3.9)
Intraparenchymal	3 (5.9)
Hydrocephalus	2 (3.9)
Leukoencephalomalacia	11 (21.6)
Other	9 (17.6)
TFUS, n(%)	
Any alteration*	16 (31.4)
Hemorrhage	8 (15.7)
Grade I GMH	6 (11.8)
Grade II GMH	—
Grade III GMH	2 (3.9)
Grade IV GMH	—
Intraparenchymal	—
Hydrocephalus	2 (3.9)
Leukoencephalomalacia	6 (11.8)
Other	3 (5.9)

* Any alteration of pathological significance.

Table 5 shows the concordance between TFUS and MRI in terms of the Papile grades assigned to the GMHs. The TFUS and MRI classifications were in agreement in 32 (62.8%) of the cases. For the detection of alterations, the PPV and NPV of TFUS were 87% and 54%, respectively. The most prevalent findings are detailed in Table 6.

**Table 5:** Correlation between TFUS and MRI for the grade of GMH.

MRI						
TFUS	Grade	0	I	II	III	IV
	0	30	5	8		
	I	2	2	2		
	II					
	III					2
	IV					

**Table 6:** PPV and NPV of TFUS for the detection of alterations, in relation to MRI.

Variable	PPV	NPV
Any alteration*	87	54
Hemorrhage	75	67
Grade I GMH	33	85
Grade II GMH	100	80
Grade III GMH	NA	100
Grade IV GMH	NA	96
Hydrocephalus	100	100
Leukoencephalomalacia	50	82

* Any alteration of pathological significance.

NA, not analyxed.

## DISCUSSION

**Figure 1:**
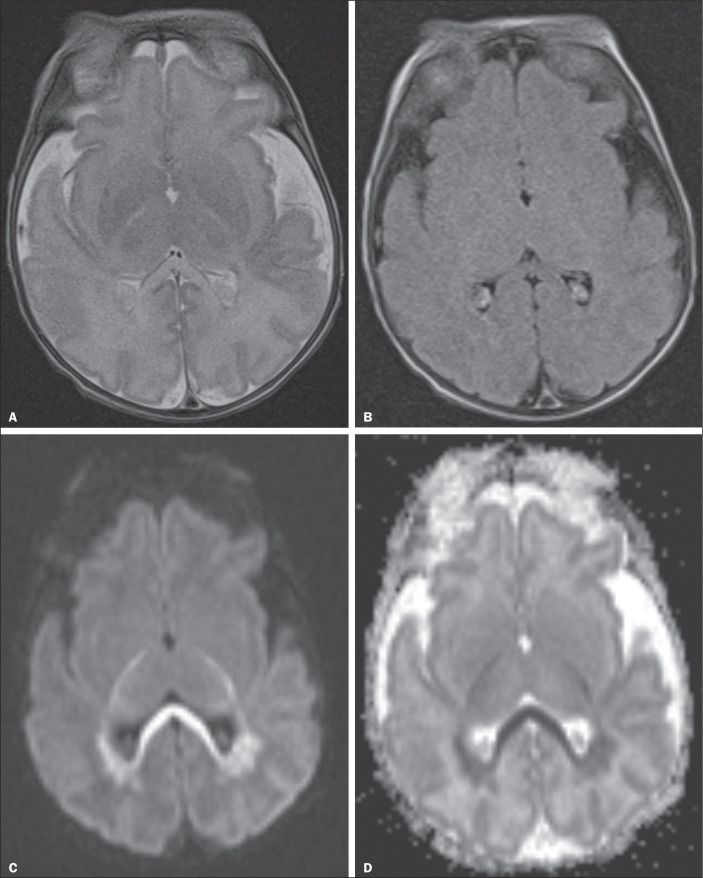
Acute periventricular leukomalacia in a preterm female newborn, born at 32 weeks of gestation, with a history of cardiopulmonary arrest and seizures, in whom TFUS had revealed no alterations. Signs of acute periventricular leukomalacia were observed on MRI: no alterations seen on a T2-weighted sequence (A) or on a fluid-attenuated inversion recovery sequence (B); and restricted diffusion seen on a diffusion-weighted sequence (C) and confirmed by apparent diffusion coefficient mapping (D).

Bedside screening with TFUS was initiated to ensure the detection of intracranial findings in very preterm infants during hospitalization^(6)^ and has now been incorporated into the daily practice of many neonatal intensive care units. Although TFUS is quite safe and accessible, the quality of the images acquired depends on the device and transducer employed, as well as on the experience of the operator, being limited by the size of the fontanelle, the angle of insonation, and the degree of signal attenuation with distance^(17)^. Those limitations often make it difficult to detect abnormalities, especially the more subtle ones, as was the case in the present study, in which TFUS had an NPV of 67% for the detection of intracranial hemorrhage, the absolute number of errors being higher for milder GMHs (Papile grades I and II). In addition, some alterations, such as those shown in Figure 1, cannot be detected on TFUS and are detected on MRI only when diffusion-weighted sequences are acquired.

According to most reports, grade I and II neonatal GMHs tend to have a low to moderate impact on long-term cognitive and motor development, a grade I hemorrhage being considered an incidental finding in most examinations, without any medium- or long-term consequences for most patients^(10,18,19)^. When we correlated the TFUS and MRI data, we found that most of the errors on TFUS were related to misclassification between grade I and II hemorrhages, as well as between grade III and IV hemorrhages.

The NPV of TFUS was lowest for grade II hemorrhages, which were classified as grade I on TFUS in two patients and were not detected on TFUS in eight. Of the 19 patients who were wrongly classified, 17 (89,5%) had hemorrhages that were graded lower on TFUS than on MRI. One possible explanation for those differences is that the hemorrhages evolved in the interval between the two examinations.

Of the 51 patients evaluated, only two (3.9%) were overdiagnosed by TFUS, which classified both as having a grade I hemorrhage, whereas MRI classified both as normal, resulting in TFUS having a low PPV (33%) for the detection of grade I hemorrhages. That has also been reported in other studies and may be related to confusion with the echogenicity of the choroid plexus, as well as the appearance of a hyperechoic nonhemorrhagic lesion in the germinal matrix^(17)^.

In a recent study of patients with neonatal intracranial hemorrhage followed for a period of five years^(10)^, those diagnosed with grade I or II hemorrhage did not develop any severe neurological sequelae, such as cerebral palsy, sensory (visual or auditory) dysfunction, mental retardation, motor retardation, and epilepsy. In that study, mild neurological sequelae, defined as mild motor delay or mild speech/language delay, were observed in only 8.8% of the patients with grade I hemorrhage and 14.2% of the patients with grade II hemorrhage, compared with 22.5% and 20.6% of the patients with grade III and IV hemorrhage, respectively, of whom 17.5% and 68.9%, respectively, developed severe neurological sequelae^(10)^. Therefore, the correct diagnosis of intracranial hemorrhages is extremely important, as is their appropriate classification, especially for grade III and IV hemorrhages, which significantly alter the prognosis.

In the present study, there were three cases in which TFUS detected no alterations and intraparenchymal hemorrhage was detected by MRI, GMH also being diagnosed in one of those cases. That underscores the difficulty of detecting peripheral and deep abnormalities by TFUS^(17)^. It is also noteworthy that TFUS had an NPV of 100% for the detection of grade III hemorrhage, as well as having a 100% PPV and NPV for the detection of hydrocephalus. Those results are probably attributable to the small sample size and the relatively low prevalence of such alterations. The small number of patients in our sample also limited the calculation of the PPV for grade III and IV hemorrhages.

When considering all of the pathological changes found on TFUS and MRI, we found TFUS to have a high overall PPV (87%), whereas its NPV was low (54%), which is, again, attributable to the limitations of the modality and the possibility that the condition of the patient worsened in the interval between the two examinations. The high PPV underscores the importance of complementing the investigation with MRI in patients in whom alterations are seen on TFUS, because MRI has greater sensitivity, allowing better definition of the location and extent of lesions, as well as the type of disease, than do TFUS and computed tomography^(20,21)^.

Our study population consisted mostly of preterm newborns, including those who were extremely preterm and had extremely low birth weights, which are the most relevant risk factors for neonatal intracranial hemorrhage^(10)^. That distribution is consistent with what is seen in many neonatal intensive care units, especially in tertiary care hospitals.

## CONCLUSION

It is extremely important that intracranial hemorrhages be characterized and classified appropriately in the neonatal period in order to provide proper care to newborns, especially those that are born preterm, with a low birth weight, or both. As a means of distinguishing between mild or moderate GMH (grade I or II) and severe GMH (grade III or IV), TFUS plays its role as a screening and follow-up examination quite well, especially in patients with the severe forms, who are often not candidates for MRI, as well as in patients with preexisting risk factors.
